# Biomechanical Evaluation of Framework Materials in All‐on‐Four Versus All‐on‐Six Prostheses: A Finite Element Study

**DOI:** 10.1002/cre2.70277

**Published:** 2026-01-26

**Authors:** Lala Cabbarova, Ali Rıza Tunçdemir, Reza Mohammadi

**Affiliations:** ^1^ Department of Prosthodontics, Faculty of Dentistry Necmettin Erbakan University Konya Turkey; ^2^ Faculty of Dentistry Necmettin Erbakan University Konya Turkey; ^3^ Faculty of Medicine, Department of Oral and Maxillofacial Diseases University of Helsinki Helsinki Finland

**Keywords:** finite element stress analysis, implant‐supported prostheses, PEEK, titanium, Trilor, Trinia, zirconia

## Abstract

**Purpose:**

The objective of this study was to biomechanically compare the All‐on‐Four and All‐on‐Six implant configurations combined with various framework materials by assessing stress distribution in peri‐implant bone, implants, and prosthetic structures using finite element analysis (FEA).

**Materials and Methods:**

This study investigated the biomechanical behavior of six different framework materials titanium, zirconia, PEEK, PEKK, Trilor and Trinia in full‐arch, implant‐supported fixed prostheses using the All‐on‐Four and All‐on‐Six concepts in a total edentulous mandible. A three dimensional finite element model of the mandible, incorporating cortical and trabecular bone as well as mucosal tissue, was developed based on CBCT data. In the All‐on‐Four configuration, two anterior implants were placed axially and two posterior implants were tilted distally at 30°. The All‐on‐Six model featured axially placed anterior implants, with posterior implants angled at 15° in the premolar and 30° in the molar regions. Multi‐unit abutments were used for all implants. Frameworks were digitally designed in a Toronto prosthesis configuration using each material, and a monolithic zirconia superstructure was applied as the veneering material. All models were subjected to a simulated vertical masticatory load of 150 N. Maximum principal stress values were assessed in the peri‐implant bone, while von Mises stress distributions were analyzed in the framework, implants, and fixation screws.

**Results:**

The highest stress accumulation was observed in the All‐on‐Four configuration, particularly around the cantilever region and distal implants. Materials with low elastic modulus (PEEK and PEKK) caused higher stress transmission to peri‐implant bone and connection components. In contrast, rigid materials (titanium and zirconia) provided a more balanced load distribution and resulted in lower stress concentrations. Glass fiber‐reinforced composite frameworks (Trilor and Trinia) remained within clinically acceptable biomechanical limits.

**Conclusion:**

The findings of this study indicated that both implant configuration and framework material properties play a critical role in the biomechanical performance and long‐term success of the prosthesis.

## Summary

- **What is known:**
  ◦ All‐on‐Four and All‐on‐Six implant configurations are widely used for full‐arch rehabilitations in edentulous mandibles.
  ◦ The choice of framework material significantly affects stress distribution in implant‐supported prostheses.
  ◦ Limited biomechanical evidence exists comparing newer composite materials (e.g., Trilor, Trinia) with traditional materials like titanium and zirconia.
- **What this study adds:**
  ◦ This finite element analysis demonstrates how framework material stiffness and implant configuration jointly influence stress on peri‐implant bone and prosthetic components.
  ◦ The findings highlight that rigid materials (titanium, zirconia) provide more favorable stress distribution, while low‐modulus polymers (PEEK, PEKK) transfer higher stress to surrounding bone and connections.

## 1 Introduction

Partial or complete edentulism has been consistently associated with adverse effects on patients' oral function, phonetics, and esthetics, which in turn contribute to significant psychosocial distress and reduced quality of life (Allen and McMillan 2003). Although conventional removable dentures have been widely used in the treatment of such patients for many years, their use is often associated with several drawbacks, including difficulties in chewing and speaking, reduced occlusal force, impaired oral sensory feedback, and compromised patient comfort (Uesugi et al. 2024). With the advent of implant‐supported fixed prostheses, novel and more effective approaches have emerged in full‐arch rehabilitation, offering improved outcomes in terms of function and aesthetics (Pjetursson et al. 2012).

Full‐arch implant therapy represents one of the most advanced contemporary techniques in implant dentistry. Fixed prostheses supported by four or six implants placed across the jaw offer multiple clinical advantages. These full‐arch implant‐supported restorations are typically performed without the need for bone grafting and have demonstrated high success rates (Rosén and Gynther 2007; Francetti et al. 2015; de Moraes et al. 2015).

The “All‐on‐Four” concept, developed by Uesugi et al. (2024), was introduced as an alternative to conventional implant protocols. This approach aims to minimize the number of implants required, thereby reducing the need for complex surgical procedures, shortening the healing period, and lowering the overall cost of treatment. In this protocol, two implants are placed axially in the anterior region, while the posterior implants are positioned at specific angles (30°–45°) (Maló et al. 2012, 2003, 2019). Several studies have demonstrated that prosthetic designs supported by four to six implants yield satisfactory functional, biological, and aesthetic outcomes (Maló et al. 2011). Nevertheless, the appropriate selection of framework materials remains essential for treatment success. Although the “All‐on‐Four” protocol is generally considered an effective treatment option, in cases with insufficient bone quality and quantity, it may be more appropriate to convert to the “All‐on‐Six” protocol by placing two additional distally tilted implants. This approach allows for extending prosthetic support up to the first molar region, thereby enhancing stability and improving load distribution (Berberi et al. 2014; Taruna et al. 2014).

The material selected for implant‐supported fixed prostheses is a critical factor in the transmission of masticatory forces and the overall durability of the prosthesis. It directly influences the load‐bearing capacity of the structure (Nazari et al. 2016). During mastication, applied forces are transmitted to the implant‐framework and implant‐bone interfaces, potentially resulting in mechanical or biological complications (Abduo and Judge 2014).

Materials used in dentistry must exhibit the necessary physical, chemical, and biological properties to ensure clinical success. A variety of framework materials are employed in implant‐supported fixed prostheses, and it is essential to comprehensively evaluate their biomechanical properties when selecting a suitable option.

Most studies on CAD‐CAM fabricated implant‐supported prostheses have focused on traditional milling materials such as titanium (Ti), zirconia, and cobalt‐chromium (Co‐Cr) alloys. However, recent advancements have introduced a range of new‐generation materials including polymer‐based composites and highly aesthetic, durable alternatives into the market. Nevertheless, comprehensive biomechanical evaluations of these novel materials remain limited in the literature (Cevik et al. 2022). PAEK (polyaryletherketone) materials, commonly referred to as PEEK (polyetheretherketone) or PEKK (polyetherketoneketone), belong to the class of engineering polymers. PEEK is a semi‐crystalline, high‐performance thermoplastic polymer. Its non‐corrosive nature, electrical non‐conductivity, radiolucency, high thermal stability, low plaque affinity, lightweight, resistance to water absorption, and excellent biocompatibility make it a highly preferred material in dentistry. Polyetherketoneketone (PEKK), on the other hand, is a new‐generation, high‐performance thermoplastic polymer that does not contain methacrylate and has attracted considerable attention due to its superior mechanical and chemical properties. Initially introduced by Bonner in 1962, PEKK was primarily used for industrial and military purposes; however, in recent years, its use as a biomaterial has significantly increased owing to its properties suitable for dental and medical applications (Huang et al. 2014; Najeeb et al. 2016; Alqurashi et al. 2021). New‐generation CAD‐CAM fiber‐reinforced composite resins (FRCs) contain a higher concentration of multidirectional glass fibers compared to conventional FRCs. Due to their low elastic modulus, high flexural strength, and shock‐absorbing properties, they are preferred as framework materials in implant‐supported fixed dental prostheses (Erkmen et al. 2011; Suzaki et al. 2020).

In the literature, significant variations have been observed regarding the direction and magnitude of masticatory and maximum bite forces. The primary reason for these differences is the physiological and functional diversity among individuals. In particular, factors such as age, gender, the presence of parafunctional habits, the segmental regions of the dental arch, and edentulous conditions play a decisive role in determining the magnitude of maximum bite forces (Linderholm and Wennström 1970).

Evaluating the biomechanical behavior of implants in the oral environment presents several challenges. In particular, analyzing the impact of different framework materials on implant behavior is often complex and time‐consuming in clinical practice. Finite element analysis (FEA) has become a valuable method in dentistry for examining stress distribution and patterns in prosthetic structures and related anatomical regions. Owing to its predictive capabilities, this method is increasingly utilized in biomechanical research (Kelkar et al. 2021).

FEA is a numerical technique commonly employed in engineering and scientific disciplines to model the behavior of complex structures and systems (David Müzel et al. 2020).

The clinical significance of this study is to demonstrate the impact of implant configuration and framework material selection on the biomechanical success and long‐term durability of full‐arch implant‐supported prostheses.

The aim of this study is to biomechanically evaluate and compare two implant placement protocols All‐on‐Four and All‐on‐Six in a fully edentulous mandibular arch, utilizing various framework materials. FEA was employed to assess stress distribution patterns within the peri‐implant bone, implants, and prosthetic components under simulated functional loading. The null hypothesis posits that neither the number of implants nor the type of framework material used in these configurations would result in significant differences in stress distribution within the bone tissue, implant assemblies, or prosthetic frameworks.

## 2 Materials and Methods

In this study, mandibular CBCT data were obtained from a completely edentulous male patient who presented to our faculty and voluntarily provided written informed consent to participate. The tomographic data acquired from the patient were utilized to construct two different implant placement configurations representing the “All‐on‐Four” and “All‐on‐Six” protocols.

CBCT imaging was performed using a NewTom Giano HR device (Bologna, Italy) with parameters of 90 kVp, 5 mA, and a voxel resolution of 0.25 mm. The obtained DICOM 3.0 files were imported into MIMICS software (Materialise, Leuven, Belgium) for anatomical segmentation of the mandible into trabecular bone, cortical bone, and mucosal tissue. Following segmentation, the data were exported in STL format to preserve anatomical geometry.

Surface optimization and refinement of the STL files were performed using Geomagic Design X 2020.0 (3D Systems, USA), after which the files were saved in STP format. The refined anatomical models were then transferred into SolidWorks 2013 (SolidWorks Corp., Waltham, MA, USA) for virtual implant planning.

**Figure 1:**
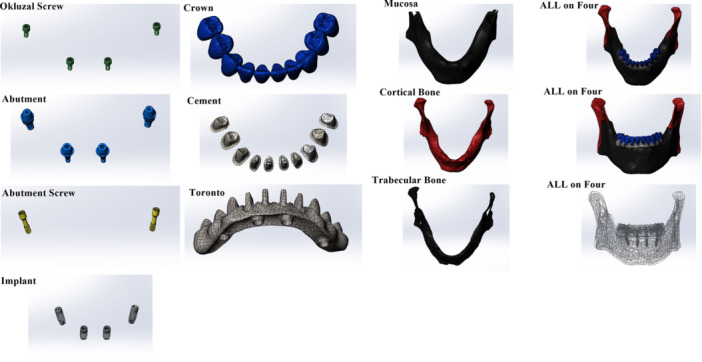
Solid models of the All‐on‐Four implant configuration illustrating the anatomical positioning of implants and prosthetic framework in a fully edentulous mandible.

**Figure 2:**
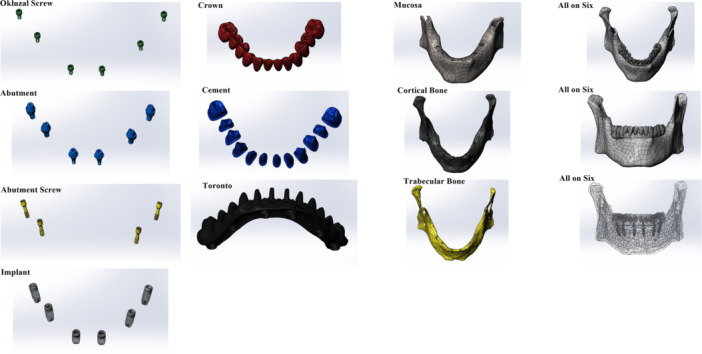
Solid models of the All‐on‐Six implant configuration illustrating the anatomical positioning of implants and prosthetic framework in a fully edentulous mandible.

In the “All‐on‐Four” implant placement protocol, the anterior implants were positioned incisors parallel and axially. In the posterior region, implants were placed in the areas corresponding to teeth #44 and #34 at a 30° distal angulation relative to the bone (Figure 1). In the “All‐on‐Six” protocol, anterior implants were also placed lateral incisors parallel and axially, while posterior implants were planned at different angulations: 15° in the areas corresponding to teeth #44 and #34, and 30° in the areas of teeth #46 and #36 (Figure 2). Straight and angled multi‐unit abutments with a gingival height of 2 mm were used for all implants. In both protocols, implants with a diameter of 3.7 mm and a length of 10 mm were utilized.

Full‐arch fixed prostheses were digitally designed for both implant configurations using six different framework materials: titanium, zirconia, PEEK, PEKK, Trilor, and Trinia. Six distinct models were developed for each implant configuration, resulting in a total of 12 prosthetic frameworks. All framework and veneering designs were created using Exocad Dental CAD 3.1 software (EXOCAD, Darmstadt, Germany). Anatomical tooth morphologies were selected from the embedded library of the software to achieve a natural appearance. The frameworks were designed in a Toronto‐style structure with monolithic zirconia veneers, and a standardized vertical height of 15 mm was adopted for all models.

A distal cantilever of 10 mm was included in the All‐on‐Four configuration, whereas no distal cantilever was used in the All‐on‐Six configuration to achieve a more favorable stress distribution strategy. The finalized STL files were re‐imported into Geomagic Design X for surface adjustments and then exported in STP format. These prosthetic designs were assembled with the mandibular model and aligned to the implant positions in the SolidWorks assembly module. For the cementation of monolithic zirconia veneers, a cement gap of 40 µm was defined and filled with resin cement. All models were then transferred to Abaqus 2020 (Dassault Systèmes, Johnston, RT, USA) for FEA.

In the prepared models, bilateral vertical occlusal forces of 150 N were applied on each side. The mandibular bone was fully fixed (encastre) in the simulation, and all movements and rotations were completely restricted. It was assumed that the implants were fully osseointegrated with the bone and that no micromovements occurred in the implant components. To define the quality of integration at the connection interfaces, frictional contact was assigned between the abutment, implant (fixture), and abutment screw, with a coefficient of friction set at 0.3. To simulate osseointegration, “tie” constraints were assigned to the interfaces between the framework and superstructure, bone and mucosa, and implant and bone. Connections between screw components were simulated by assigning torque properties. The screw torque moment is expressed by the following equation:

T=K×D×F

where *T* represents the screw torque moment (Ncm); *D* is the screw diameter (m); *F* is the screw preload (N); and *K* denotes the screw factor or torque coefficient (generally taken as 0.2). Using this equation, the preload value of the screw can be calculated when a specific tightening torque is applied to the abutment screw.

In this study, a tightening torque of 25 Ncm was applied, resulting in a calculated screw preload of 781 N. Following this procedure, stress analyses were performed, and the obtained stress values were reported as von Mises stress (vMS).

Under simulated loading conditions, the maximum vMS values in the implants, abutments, abutment screws, prosthetic screws, frameworks, bone, and different framework materials were evaluated and analyzed.

The material properties for all components included in the study were thoroughly defined (Table 1). These specified properties are based on the characteristic mechanical performance of each material as reported in the literature.

**Table 1:** Material properties assigned to the model (Topcu Ersöz and Mumcu 2022; Ajaj al‐Kordy and AL‐Saadi 2023; Martani 2023; Lee et al. 2017; Chen et al. 2023; Erdoğdu et al. 2024).

Material	Young's modulus (E MPa)	Poisson's ratio (ν)
Cortical bone	13,700	0.30
Trabecular bone	1370	0.30
Mucosa	2.8	0.40
Titanium (implant, abutment, screw)	110,000	0.30
Titanium (framework)	110,000	0.28
Zirconia (framework, crown)	210,000	0.30
PEEK (framework)	4200	0.36
PEKK (framework)	5100	0.40
Trinia (framework)	18,500	0.30
Trilor (framework)	26,000	0.30
Resin cement	5100	0.27

## 3 Results

### 3.1 Maximum Von Mises Stress Values in Implants

According to the FEA results obtained in this study, the maximum vMS values in the implants varied depending on both the framework material used and the implant configuration. In the All‐on‐Four configuration, stress accumulation was more pronounced, particularly around the distal implants and in the cantilever region.

When low elastic modulus materials such as PEEK and PEKK were used, higher stress values were observed around the implants. This can be attributed to the flexible nature of these materials, which allows for greater transmission of occlusal forces to the implants and peri‐implant bone. The highest stress values were recorded in the All‐on‐Four models with PEEK and PEKK frameworks.

**Figure 3:**
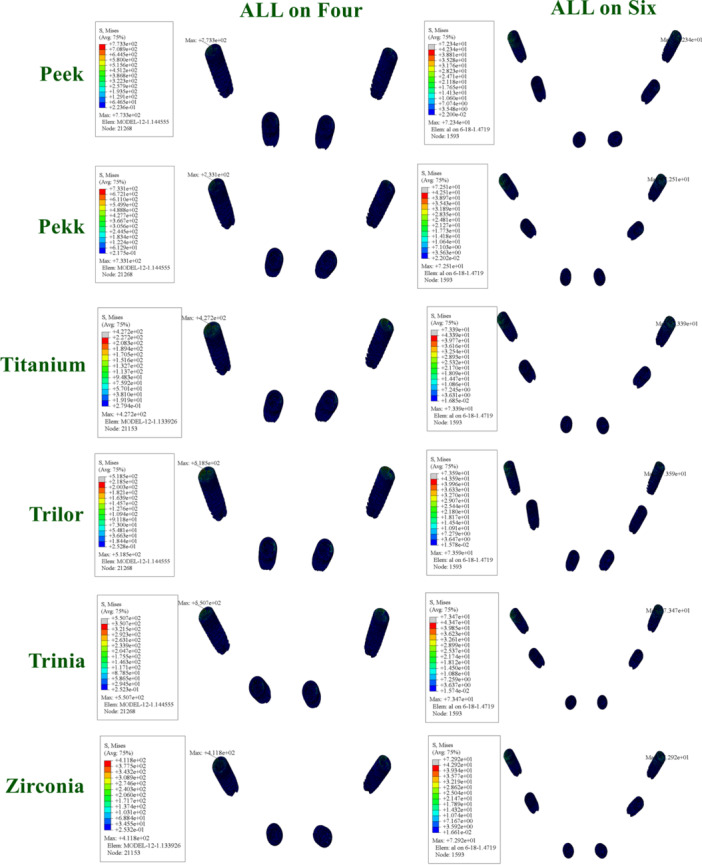
Distribution and maximum values of von Mises stress in implants under functional loading conditions.

In contrast, the All‐on‐Six configuration demonstrated a more homogeneous stress distribution, with reduced load accumulation in the distal implants. In this configuration, the load per implant decreased, providing a more balanced biomechanical environment. The lowest stress values were achieved with rigid materials (titanium and zirconia) in the All‐on‐Six models (Figure 3).

### 3.2 Von Mises Stress Values in Abutments

When examining the vMS values in the abutments, higher stress accumulation was observed in the posterior regions of the All‐on‐Four configuration. This finding can be explained by the tilted placement of the posterior implants and the cantilever effect.

In models using PEEK and PEKK frameworks, stress values in the abutments were higher compared to other materials. This can be attributed to the lower rigidity of these materials, which allows greater deformation and facilitates stress transfer to the abutments.

**Figure 4:**
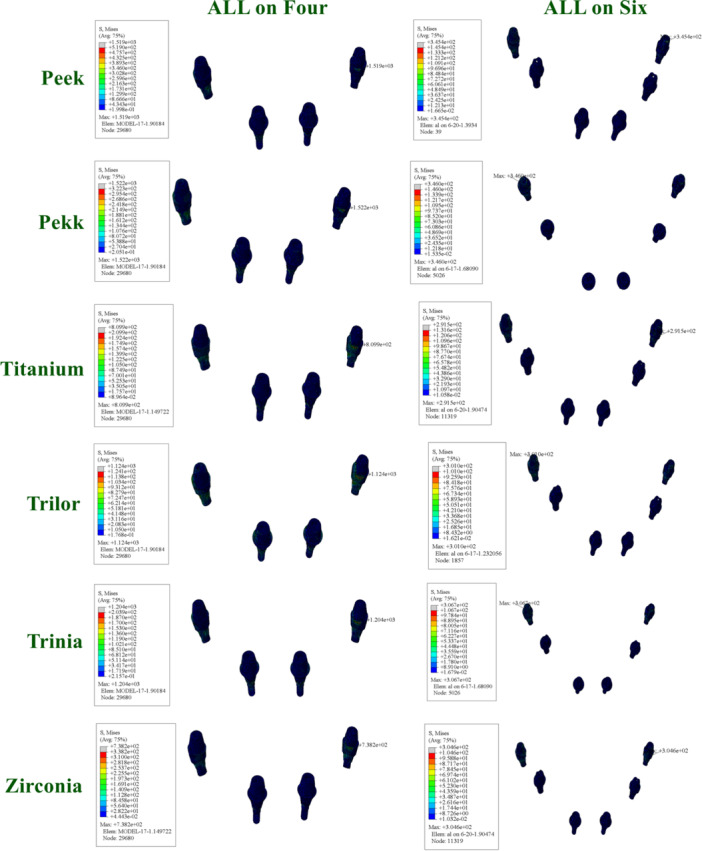
Distribution and maximum values of von Mises stress in abutments under functional loading conditions.

In the All‐on‐Six configuration, the stress values in the abutments were significantly reduced. Rigid framework materials (titanium and zirconia) exhibited the lowest stress levels in the abutments, while FRC materials (Trilor and Trinia) presented moderate stress values (Figure 4).

### 3.3 Von Mises Stress Values in Abutment Screws and Prosthetic Screws

The vMS values in the abutment screws and prosthetic screws varied depending on the framework material and implant configuration. Notably, higher stress concentrations were recorded in the screws in the All‐on‐Four configuration.

The highest stress values in the abutment and prosthetic screws were observed in models with PEEK and PEKK frameworks. This result is linked to the flexible frameworks directing more load towards the connection components. Conversely, significantly lower stress values were found in the screws of models using titanium and zirconia frameworks.

**Figure 5:**
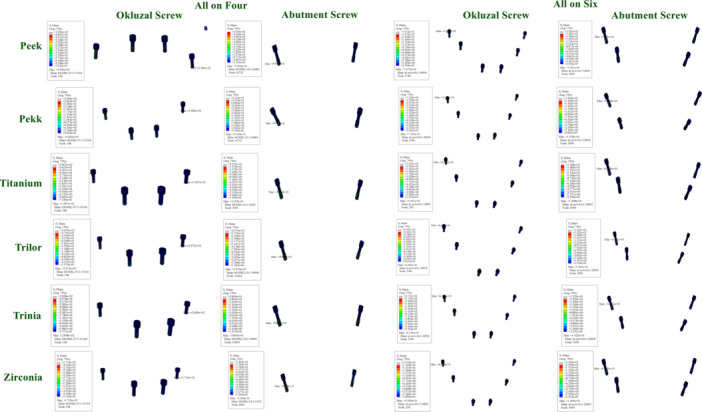
Distribution and maximum values of von Mises stress in okluzal screws and abutment screws under functional loading conditions.

In the All‐on‐Six configuration, the increased number of implants allowed for a more balanced distribution of stresses in the abutment and prosthetic screws. Especially in rigid frameworks, this provided a mechanical advantage in terms of the safety of connection components (Figure 5).

### 3.4 Maximum Von Mises Stress Values in Prosthetic Frameworks

In the All‐on‐Four configuration, the following values were found for the Toronto prosthesis: PEEK (805.7 MPa), PEKK (692.3 MPa), titanium (1117 MPa), Trilor (818.2 MPa), Trinia (794.5 MPa), and zirconia (1372 MPa). Trinia exhibited lower values compared to PEEK.

**Figure 6:**
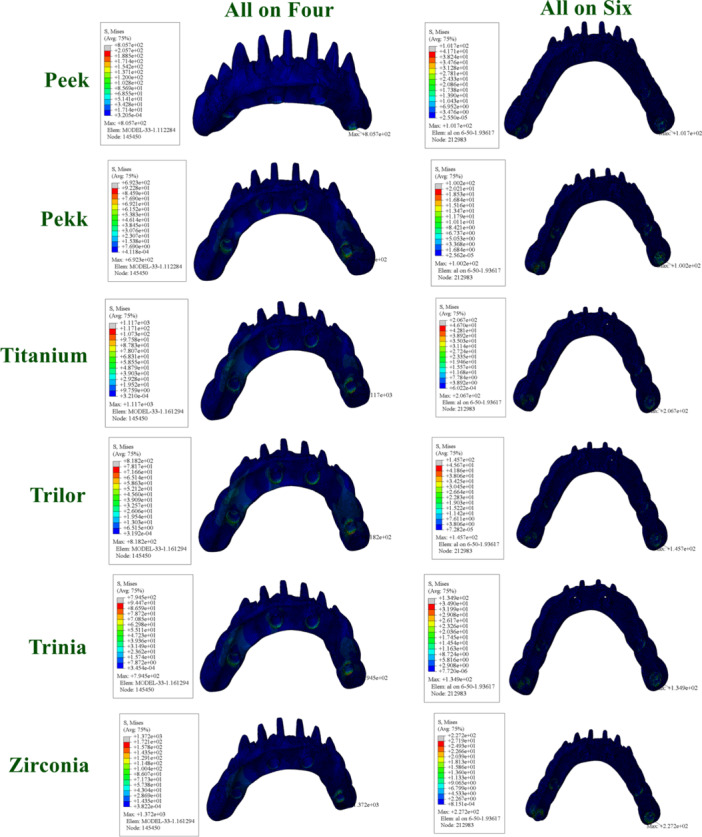
Distribution and maximum values of von Mises stress in prosthetic frameworks under functional loading conditions.

In the All‐on‐Six configuration, following values were found for the Toronto prosthesis: PEEK (101.7 MPa), PEKK (100.2 MPa), titanium (206.7 MPa), Trilor (145.7 MPa), Trinia (134.9 MPa), and zirconia (227.2 MPa) (Figure 6).

### 3.5 Maximum Von Mises Stress Values in Cortical Bone

The vMS values in the cortical bone varied significantly depending on the framework material and implant placement protocol.

In the All‐on‐Four configuration, high stress accumulation was detected particularly around the distal implants in the cortical bone. This stress concentration was even higher in models with PEEK and PEKK frameworks, whereas more controlled stress distribution was observed with titanium and zirconia frameworks.

**Figure 7:**
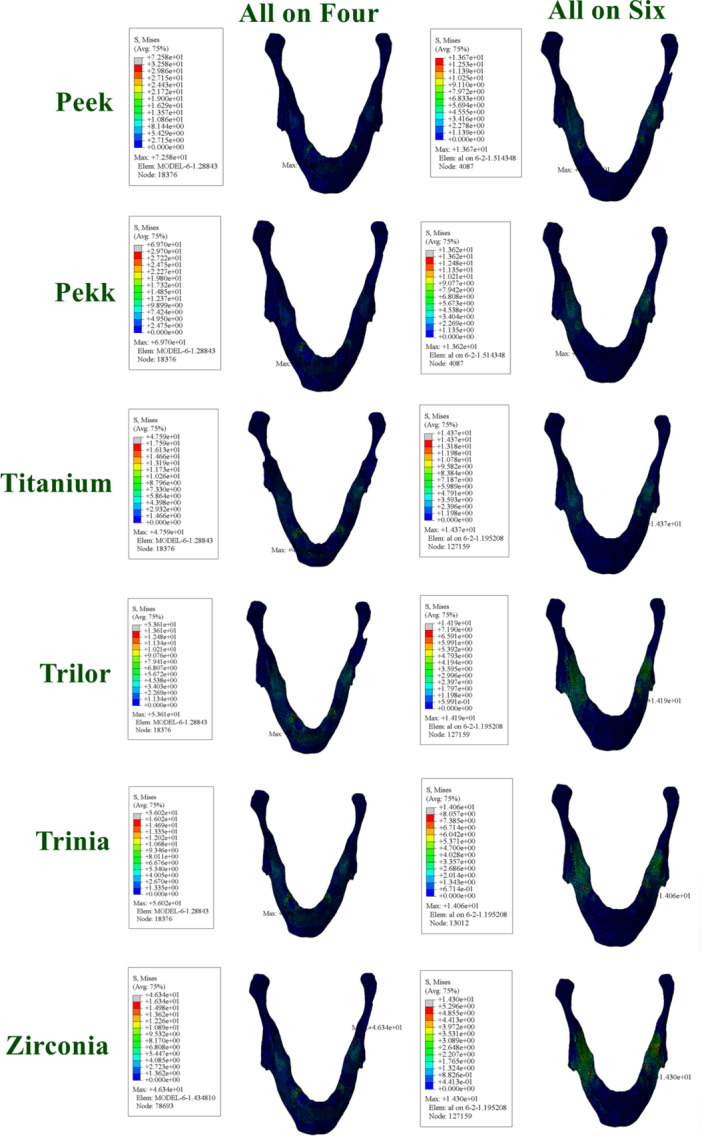
Distribution and maximum values of von Mises stress in cortical bone under functional loading conditions.

In the All‐on‐Six configuration, the stress levels in the cortical bone were generally lower. This finding is attributed to the more balanced load sharing resulting from the increased number of implants. FRC frameworks also presented clinically acceptable stress levels in the cortical bone (Figure 7).

### 3.6 Maximum Von Mises Stress Values in Cancellous Bone

The stress values observed in the cancellous bone were generally lower compared to those in the cortical bone but exhibited similar distribution patterns.

In the All‐on‐Four configuration, higher stress accumulation was observed in the distal regions of the cancellous bone. The highest stress values in these regions were recorded in models with PEEK and PEKK frameworks.

**Figure 8:**
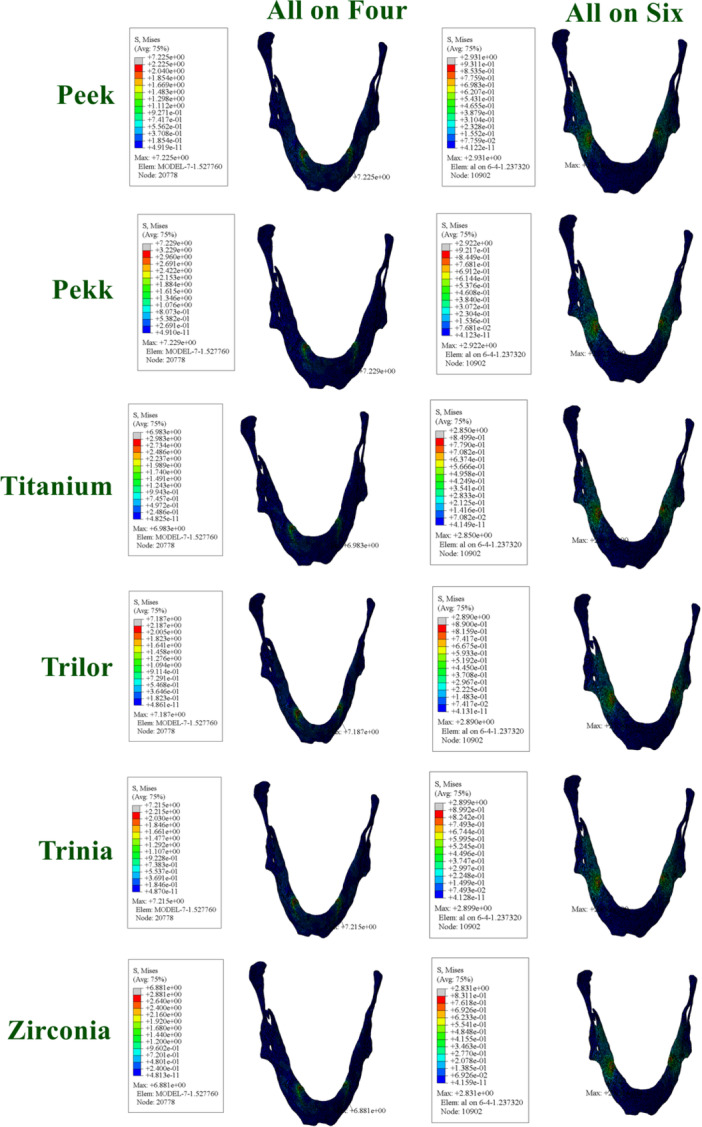
Distribution and maximum values of von Mises stress in cancellous bone under functional loading conditions.

In the All‐on‐Six configuration, the stress in the cancellous bone was more homogeneously distributed and generally lower. Rigid frameworks minimized stress levels in the cancellous bone, while FRC frameworks provided moderate stress distribution, yielding biomechanically satisfactory outcomes (Figure 8).

### 3.7 Maximum Von Mises Stress Values in Resin Cement

In the All‐on‐Four configuration, the highest stress values were observed with Peek (131.4 MPa) and Pekk (119.1 MPa) frameworks. In contrast, zirconia exhibited the lowest stress value on the cement layer (12.21 MPa), followed by titanium (16.43 MPa). Lower stress values were observed for Trilor (40.59 MPa) and Trinia (52.36 MPa), suggesting that these materials exhibit a balanced behavior between rigidity and flexibility.

**Figure 9:**
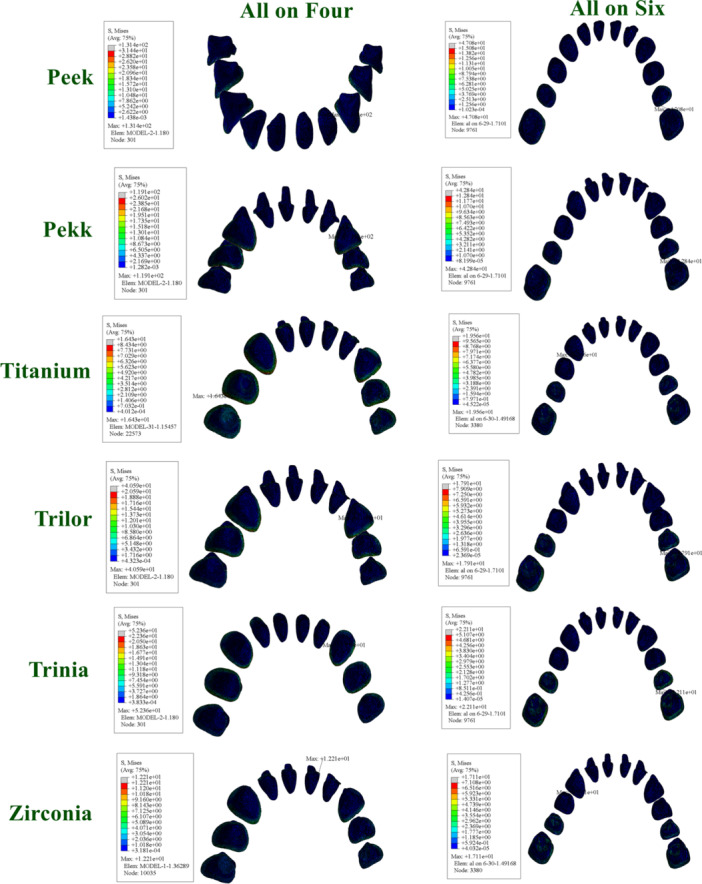
Distribution and maximum values of von Mises stress in resin cement under functional loading conditions.

In the All‐on‐Six configuration, the highest stress values were found with Peek (47.08 MPa) and Pekk (42.84 MPa) frameworks. Titanium (19.56 MPa) and zirconia (17.11 MPa) exhibited the lowest stress values. Trilor (17.91) and Trinia (22.11) exhibited stress values comparable to those of titanium and zirconia (Figure 9).

### 3.8 Maximum Von Mises Stress Values in Crown

In the All‐on‐Four configuration, the highest stress values were found with Peek (47.08 MPa) and Pekk (42.84 MPa) frameworks. Although these values are lower than those recorded in the All‐on‐Four configuration, they still reflect the higher stress transmission associated with polymer‐based materials due to their lower elastic modulus and greater deformation capacity. Titanium (19.56 MPa) and zirconia (17.11 MPa) exhibited the lowest stress values, indicating their superior ability to distribute loads and minimize stress concentrations at the cement interface. Lower stress values were observed for Trilor (17.91 MPa) and Trinia (22.11 MPa), suggesting that these materials exhibit a balanced behavior between rigidity and flexibility.

**Figure 10:**
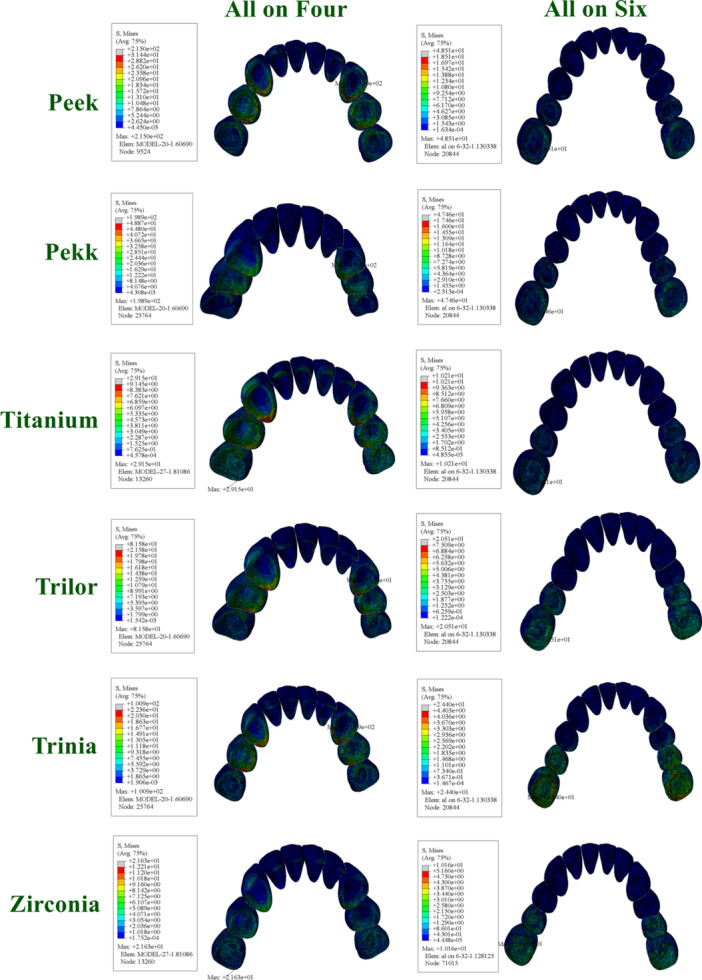
Distribution and maximum values of von Mises stress in crown under functional loading conditions.

Similarly, in the All‐on‐Six configuration, the highest stress values on crowns were observed with Peek (48.51 MPa) and Pekk (47.46 MPa) frameworks. Trilor (20.51 MPa) and Trinia (24.40 MPa) exhibited lower stress levels, consistent with their balanced elastic properties. Zirconia (10.16 MPa) and titanium (10.21 MPa) demonstrated the lowest stress levels on crowns (Figure 10).

## 4 Discussion

In this study, the biomechanical behaviors of different framework materials (titanium, zirconia, PEEK, PEKK, Trilor, and Trinia) used in All‐on‐Four and All‐on‐Six implant configurations placed in a completely edentulous mandibular arch were thoroughly evaluated using the FEA method. The stress distributions in the implants, abutments, abutment screws, prosthetic screws, and surrounding bone tissues, as well as the fatigue performances of the framework materials, were analyzed. The null hypothesis was rejected, as it was demonstrated that both implant configuration and framework material significantly affect the stress distribution in implants, implant components, and surrounding bone tissue.

Accurately modeling the magnitude, direction, and location of applied loads in FEA to reflect clinical conditions enables more consistent and predictable insights into the mechanical behavior of tissues and prosthetic structures. For the success of implant‐supported fixed restorations, it is essential to ensure proper distribution of occlusal loads on the prosthetic framework.

Morneburg and Pröschel (2002) reported bite forces ranging between 200 and 300 N in individuals with fixed prostheses, while Müller et al. (2012) measured forces within the same range for implant‐supported prostheses. Bhering et al. (2016) applied a 150 N load in All‐on‐Four and All‐on‐Six designs, whereas Topcu Ersöz and Mumcu (2022) used a 200 N load. Özdemir Doğan et al. (2014) applied a 300 N oblique load to evaluate the cantilever effect. Kumari et al. (2020) assessed multiple loading scenarios targeting different regions (anterior, posterior, and lateral) and analyzed stress distribution in implants and surrounding tissues in detail.

In this study, considering previous research, a bilateral vertical load of 150 N was applied to the posterior region. This loading strategy aimed to simulate clinical masticatory forces more realistically and contributed to a more accurate analysis of stress distribution.

The results demonstrated that both the elastic modulus of the framework material and the implant configuration have significant effects on stress distribution and mechanical stability in implant‐supported full‐arch restorations. In particular, in the All‐on‐Four configuration, stress accumulation was markedly higher in the distal implants and cantilever regions. This finding aligns with previous reports indicating that increased cantilever length and reduced implant number elevate stress concentrations in the peri‐implant bone, thereby increasing the potential risk of bone resorption (Silva et al. 2010; Behnaz et al. 2015).

When evaluating the framework materials, it was observed that titanium and zirconia frameworks generated the lowest vMS values due to their high elastic modulus. The rigid nature of these materials enabled more homogeneous load distribution, reduced stress transfer to peri‐implant tissues, and contributed to the protection of connection components. These findings are consistent with results reported by Bhering et al. (2016) and Sailer et al. (2012), which highlighted that frameworks with higher elastic modulus reduce stress on implants and surrounding bone.

Conversely, restorations with PEEK and PEKK frameworks exhibited higher stress values in the implants and bone tissues due to their flexible nature. Owing to their lower elastic modulus, these materials transmitted a greater portion of the load to the peri‐implant region, thereby potentially increasing the risk of biological complications. Similarly, higher stress concentrations were also observed in the abutment and prosthetic screws in these models, which may pose a potential disadvantage by increasing the risk of mechanical complications (Lee et al. 2017; Dayan and Geckili 2021).

FRC materials (Trilor and Trinia), while not as rigid as titanium and zirconia, provided a more balanced stress distribution compared to PEEK and PEKK. Their intermediate elastic properties allowed for a balance between rigidity and flexibility, suggesting that they may be suitable alternatives in clinical applications from a biomechanical perspective. These findings align with the literature suggesting that FRC contribute to reducing mechanical stress in implant‐supported restorations due to their shock‐absorbing capacities (Passaretti et al. 2018; De Giorgis et al. 2024).

These findings indicate that more rigid framework materials enhance long‐term mechanical durability, thereby supporting the overall success of implants and prosthetic components (Bhering et al. 2016; Jacques et al. 2009; Assunção et al. 2010).

Stress distribution on the abutments also plays a critical role in implant dentistry. The literature indicates that fatigue‐related fractures typically occur at the weakest point of the system. In our study, the highest stress values were observed on the abutments across both configurations and all framework materials, with the initial deformation occurring on the abutments in the posterior region (López‐Píriz et al. 2019; Lauritano et al. 2020).

High stress values observed in the occlusal screw indicate increased functional loading, which may lead to complications such as preload loss, screw loosening, and potential fractures. As shown in various studies (Bozkaya and Müftü 2003; Ricciardi Coppedê et al. 2013; Shin et al. 2014), reduced preload and micromovements under cyclic occlusal loads can compromise joint stability. Therefore, proper preload application, precise torque control, and appropriate screw and connection design are critical to ensure long‐term mechanical stability and prevent mechanical failures in implant‐supported prostheses.

The cement and crown stress values found in our study were consistent with the other results, indicating that polymer‐based materials transmitted higher stresses due to their low elastic modulus, whereas Trilor and Trinia demonstrated acceptable biological stress levels, and zirconia and titanium exhibited lower values due to their rigid structures.

When assessing the forces transmitted to the bone, σMax and σMin values are analyzed. In cortical bone, compressive forces exceeding 170–190 MPa and tensile forces surpassing 100–130 MPa are considered critical thresholds that may increase the risk of bone resorption due to excessive stress accumulation (Baggi et al. 2013). Across all analyses, higher stress concentrations were observed in the alveolar crest region. The values obtained in the study were found to be below the thresholds reported for bone resorption.

From an implant configuration perspective, it was determined that the All‐on‐Six, due to the increased number of implants, achieved a more homogeneous stress distribution, resulting in generally lower stress levels in the peri‐implant bone and connection components. Moreover, in the All‐on‐Six configuration, similar stress values were observed across all framework materials. This outcome supports existing literature indicating that increasing the number of implants, especially in the posterior region, reduces the cantilever effect, optimizes load sharing, and enhances biomechanical stability (Silva et al. 2010; Pandey et al. 2023).

In conclusion, the findings of this study demonstrate that the selection of framework material and implant configuration play critical roles in the biomechanical success of implant‐supported full‐arch restorations. Utilizing rigid materials such as titanium and zirconia and increasing the number of implants (All‐on‐Six) optimize stress distribution and improve fatigue performance, thereby supporting long‐term clinical success.

Future clinical studies and long‐term follow‐up data will further strengthen these findings. Additionally, evaluating different patient groups, various masticatory forces, and diverse occlusal loading scenarios may provide more precise insights for personalized biomechanical planning.

In the modeling phase, it was assumed that all components were flawlessly joined in different configurations, and the material properties were considered isotropic and linear. However, the use of FEA may not fully replicate the complex biomechanical interactions present in in vivo conditions, which constitutes a major limitation of this study. Therefore, it is crucial that future studies validate these findings through in vivo experiments to enhance their clinical relevance and reliability.

## 5 Conclusion

This study evaluated the biomechanical behaviors of All‐on‐Four and All‐on‐Six implant‐supported full‐arch prostheses with different framework materials using FEA. The results showed that increasing the number of implants and using rigid framework materials such as titanium and zirconia improved stress distribution and enhanced fatigue performance. All‐on‐Six configurations provided more homogeneous stress distribution, reducing the risk of bone resorption and mechanical complications compared to the All‐on‐Four concept. Framework materials with high elastic modulus, particularly titanium and zirconia, minimized stress on implants and surrounding bone, supporting long‐term success. In contrast, polymer‐based materials like PEEK and PEKK showed higher stress transmission to peri‐implant tissues and lower fatigue resistance. FRC (Trilor and Trinia) offered a balance between rigidity and flexibility, presenting as viable alternatives. In conclusion, selecting an appropriate implant configuration and framework material is crucial to optimize biomechanical performance and ensure long‐term clinical success in full‐arch restorations.

## Author Contributions

Lala Cabbarova, Ali Rıza Tunçdemir, and Reza Mohammadi participated in designing the study, generating and gathering the data for the study, reviewed the pertinent raw data on which the results and conclusions of this study are based, approved the final version of this paper, and guarantees that all individuals who meet the Journal's authorship criteria are included as authors of this paper. Reza Mohammadi participated in the analysis of the data and has had access to all the raw data of the study.

## Funding

The authors received no specific funding for this work.

## Ethics Statement

This study was conducted in accordance with the ethical principles of the Declaration of Helsinki. The CBCT data were used in compliance with ethical standards. Ethical approval for the study was granted by the Ethics Committee of the Faculty of Dentistry, Necmettin Erbakan University, Konya, Turkey (Approval ID: 23133), with unanimous agreement of the committee members.

## Consent

Written informed consent was obtained from an edentulous patient prior to CBCT imaging.

## Conflicts of Interest

The authors declare no conflicts of interest.

## Data Availability

The datasets used and/or analyzed during the current study are available from the corresponding author on reasonable request.

## References

[cre270277-bib-0001] Abduo, J. , and R. Judge . 2014. “Implications of Implant Framework Misfit: A Systematic Review of Biomechanical Sequelae.” International Journal of Oral & Maxillofacial Implants 29: 608–621. 24818199 10.11607/jomi.3418

[cre270277-bib-0002] Ajaj al‐Kordy, N. M. T. , and M. H. AL‐Saadi . 2023. “Finite Element Study of Stress Distribution With Tooth‐Supported Mandibular Overdenture Retained by Ball Attachments or Resilient Telescopic Crowns.” European Journal of Dentistry 17: 539–547. 36351452 10.1055/s-0042-1749363 PMC10329555

[cre270277-bib-0003] Allen, P. F. , and A. S. McMillan . 2003. “A Review of the Functional and Psychosocial Outcomes of Edentulousness Treated With Complete Replacement Dentures.” Journal of the Canadian Dental Association 69: 662. 14611716

[cre270277-bib-0004] Alqurashi, H. , Z. Khurshid , A. U. Y. Syed , S. Rashid Habib , D. Rokaya , and M. S. Zafar . 2021. “Polyetherketoneketone (PEKK): An Emerging Biomaterial for Oral Implants and Dental Prostheses.” Journal of Advanced Research 28: 87–95. 33384878 10.1016/j.jare.2020.09.004 PMC7770505

[cre270277-bib-0005] Assunção, W. G. , É. A. Gomes , V. A. R. Barão , J. A. Delben , L. F. Tabata , and E. A. C. de Sousa . 2010. “Effect of Superstructure Materials and Misfit on Stress Distribution in a Single Implant‐Supported Prosthesis.” Journal of Craniofacial Surgery 21: 689–695. 20485030 10.1097/SCS.0b013e3181d7f2e5

[cre270277-bib-0006] Baggi, L. , S. Pastore , M. Di Girolamo , and G. Vairo . 2013. “Implant‐Bone Load Transfer Mechanisms in Complete‐Arch Prostheses Supported by Four Implants: A Three‐Dimensional Finite Element Approach.” Journal of Prosthetic Dentistry 109: 9–21. 23328192 10.1016/S0022-3913(13)60004-9

[cre270277-bib-0007] Behnaz, E. , M. Ramin , S. Abbasi , M. A. Pouya , and F. Mahmood . 2015. “The Effect of Implant Angulation and Splinting on Stress Distribution in Implant Body and Supporting Bone: A Finite Element Analysis.” European Journal of Dentistry 9: 311–318. 26430356 10.4103/1305-7456.163235 PMC4569979

[cre270277-bib-0008] Berberi, A. N. , J. M. Sabbagh , M. N. Aboushelib , Z. F. Noujeim , and Z. A. Salameh . 2014. “A 5‐year Comparison of Marginal Bone Level Following Immediate Loading of Single‐Tooth Implants Placed in Healed Alveolar Ridges and Extraction Sockets in the Maxilla.” Frontiers in Physiology 5: 29. 24550840 10.3389/fphys.2014.00029 PMC3908518

[cre270277-bib-0009] Bhering, C. L. B. , M. F. Mesquita , D. T. Kemmoku , P. Y. Noritomi , R. L. X. Consani , and V. A. R. Barão . 2016. “Comparison Between All‐on‐Four and All‐on‐Six Treatment Concepts and Framework Material on Stress Distribution in Atrophic Maxilla: A Prototyping Guided 3D‐FEA Study.” Materials Science and Engineering: C 69: 715–725. 27612765 10.1016/j.msec.2016.07.059

[cre270277-bib-0010] Bozkaya, D. , and S. Müftü . 2003. “Mechanics of the Tapered Interference Fit in Dental Implants.” Journal of Biomechanics 36: 1649–1658. 14522206 10.1016/s0021-9290(03)00177-5

[cre270277-bib-0011] Cevik, P. , M. Schimmel , and B. Yilmaz . 2022. “New Generation CAD‐CAM Materials for Implant‐Supported Definitive Frameworks Fabricated by Using Subtractive Technologies.” BioMed Research International 2022, no. 1: 3074182. 35281596 10.1155/2022/3074182 PMC8906986

[cre270277-bib-0012] Chen, S. , X. Hong , Z. Ye , et al. 2023. “The Effect of Root Canal Treatment and Post‐Crown Restorations on Stress Distribution in Teeth With Periapical Periodontitis: A Finite Element Analysis.” BMC Oral Health 23: 973. 38057755 10.1186/s12903-023-03612-9 PMC10701996

[cre270277-bib-0013] David Müzel, S. , E. P. Bonhin , N. M. Guimarães , and E. S. Guidi . 2020. “Application of the Finite Element Method in the Analysis of Composite Materials: A Review.” Polymers 12: 818. 32260389 10.3390/polym12040818 PMC7240738

[cre270277-bib-0014] Dayan, S. C. , and O. Geckili . 2021. “The Influence of Framework Material on Stress Distribution in Maxillary Complete‐Arch Fixed Prostheses Supported by Four Dental Implants: A Three‐Dimensional Finite Element Analysis.” Computer Methods in Biomechanics and Biomedical Engineering 24: 1606–1617. 33798003 10.1080/10255842.2021.1903450

[cre270277-bib-0015] Erdoğdu, M. , M. G. Demirel , R. Mohammadi , N. Güntekin , and M. Ghanbarzadeh Chaleshtori . 2024. “Influence of Framework Material and Abutment Configuration on Fatigue Performance in Dental Implant Systems: A Finite Element Analysis.” Medicina 60: 1463. 39336504 10.3390/medicina60091463 PMC11433853

[cre270277-bib-0016] Erkmen, E. , G. Meriç , A. Kurt , Y. Tunç , and A. Eser . 2011. “Biomechanical Comparison of Implant Retained Fixed Partial Dentures With Fiber Reinforced Composite Versus Conventional Metal Frameworks: A 3D FEA Study.” Journal of the Mechanical Behavior of Biomedical Materials 4: 107–116. 21094484 10.1016/j.jmbbm.2010.09.011

[cre270277-bib-0017] Francetti, L. , A. Rodolfi , B. Barbaro , S. Taschieri , N. Cavalli , and S. Corbella . 2015. “Implant Success Rates in Full‐Arch Rehabilitations Supported by Upright and Tilted Implants: A Retrospective Investigation With up to Five Years of Follow‐up.” Journal of Periodontal & Implant Science 45: 210. 26734491 10.5051/jpis.2015.45.6.210 PMC4698947

[cre270277-bib-0018] De Giorgis, L. , P. Pesce , F. Barberis , et al. 2024. “Fiber‐Reinforced Composites for Full‐Arch Implant‐Supported Rehabilitations: An In Vitro Study.” Journal of Clinical Medicine 13: 2060. 38610826 10.3390/jcm13072060 PMC11012982

[cre270277-bib-0019] Huang, B. , J. Qian , G. Wang , and M. Cai . 2014. “Synthesis and Properties of Novel Copolymers of Poly(Ether Ketone Diphenyl Ketone Ether Ketone Ketone) and Poly(Ether Amide Ether Amide Ether Ketone Ketone).” Polymer Engineering & Science 54: 1757–1764.

[cre270277-bib-0020] Jacques, L. B. , M. S. Moura , V. Suedam , E. A. C. Souza , and J. H. Rubo . 2009. “Effect of Cantilever Length and Framework Alloy on the Stress Distribution of Mandibular‐Cantilevered Implant‐Supported Prostheses.” Clinical Oral Implants Research 20: 737–741. 19489929 10.1111/j.1600-0501.2009.01712.x

[cre270277-bib-0021] Kelkar, K. C. , V. Bhat , and C. Hegde . 2021. “Finite Element Analysis of the Effect of Framework Materials at the Bone‐Implant Interface in the All‐on‐Four Implant System.” Dental Research Journal 18: 1. 34084288 PMC8122683

[cre270277-bib-0022] Kumari, A. , P. Malhotra , S. Phogat , B. Yadav , J. Yadav , and S. Phukela . 2020. “A Finite Element Analysis to Study the Stress Distribution on Distal Implants in an All‐on‐Four Situation in Atrophic Maxilla as Affected by the Tilt of the Implants and Varying Cantilever Lengths.” Journal of Indian Prosthodontic Society 20: 409. 33487969 10.4103/jips.jips_70_20 PMC7814689

[cre270277-bib-0023] Lauritano, D. , G. Moreo , A. Lucchese , C. Viganoni , L. Limongelli , and F. Carinci . 2020. “The Impact of Implant–Abutment Connection on Clinical Outcomes and Microbial Colonization: A Narrative Review.” Materials 13: 1131. 32138368 10.3390/ma13051131 PMC7085009

[cre270277-bib-0024] Lee, K.‐S. , S. W. Shin , S. P. Lee , J. E. Kim , J. H. Kim , and J. Y. Lee . 2017. “Comparative Evaluation of a Four‐Implant–Supported Polyetherketoneketone Framework Prosthesis: A Three‐Dimensional Finite Element Analysis Based on Cone Beam Computed Tomography and Computer‐Aided Design.” International Journal of Prosthodontics 30: 581–585. 29095963 10.11607/ijp.5369

[cre270277-bib-0025] Linderholm, H. , and A. Wennström . 1970. “Isometric Bite Force and Its Relation to General Muscle Forge and Body Build.” Acta Odontologica Scandinavica 28: 679–689. 5275811 10.3109/00016357009058590

[cre270277-bib-0026] López‐Píriz, R. , B. Cabal , L. Goyos‐Ball , et al. 2019. “Current State‐of‐the‐Art and Future Perspectives of the Three Main Modern Implant‐Dentistry Concerns: Aesthetic Requirements, Mechanical Properties, and Peri‐Implantitis Prevention.” Journal of Biomedical Materials Research. Part A 107: 1466–1475. 30786152 10.1002/jbm.a.36661

[cre270277-bib-0027] Maló, P. , M. de Araújo Nobre , A. Lopes , A. Ferro , and M. Nunes . 2019. “The All‐on‐4 Concept for Full‐Arch Rehabilitation of the Edentulous Maxillae: A Longitudinal Study With 5‐13 Years of Follow‐up.” Clinical Implant Dentistry and Related Research 21: 538–549. 30924250 10.1111/cid.12771

[cre270277-bib-0028] Maló, P. , M. de Araújo Nobre , A. Lopes , C. Francischone , and M. Rigolizzo . 2012. “‘All‐on‐4’ Immediate‐Function Concept for Completely Edentulous Maxillae: A Clinical Report on the Medium (3 Years) and Long‐Term (5 Years) Outcomes.” Clinical Implant Dentistry and Related Research 14: e139–e150. 22008153 10.1111/j.1708-8208.2011.00395.x

[cre270277-bib-0029] Maló, P. , Md Nobre , and A. Lopes . 2011. “The Rehabilitation of Completely Edentulous Maxillae With Different Degrees of Resorption With Four or More Immediately Loaded Implants: A 5‐year Retrospective Study and a New Classification.” European Journal of Oral Implantology 4: 227–243. 22043467

[cre270277-bib-0030] Maló, P. , B. Rangert , and M. Nobre . 2003. “‘All‐on‐Four’ Immediate‐Function Concept With Brånemark System® Implants for Completely Edentulous Mandibles: A Retrospective Clinical Study.” Clinical Implant Dentistry and Related Research 5: 2–9. 12691645 10.1111/j.1708-8208.2003.tb00010.x

[cre270277-bib-0031] Martani, N. S. , and B. N. Hadi . 2023. “Investigating The Effect of Trinia and Zirconia Implant Supported Fixed Partial Denture on Stress Distribution in Peripheral Bone: A Three Dimensional Finite Element Modeling.” Eurasian Journal of Science and Engineering 9, no. 1: 252–261.

[cre270277-bib-0032] de Moraes, P. H. , S. Olate , A. Lauria , L. Asprino , M. de Moraes , and J. R. de Albergaria‐Barbosa . 2015. “8‐10 Year Follow‐up Survival of Dental Implants in Maxillae With or Without Autogenous Bone Graft Reconstruction.” International Journal of Clinical and Experimental Medicine 8: 19282–19289. 26770565 PMC4694465

[cre270277-bib-0033] Morneburg, T. R. , and P. A. Pröschel . 2002. “Measurement of Masticatory Forces and Implant Loads: A Methodologic Clinical Study.” International Journal of Prosthodontics 15: 20–27. 11887595

[cre270277-bib-0034] Müller, F. , M. Hernandez , L. Grütter , L. Aracil‐Kessler , D. Weingart , and M. Schimmel . 2012. “Masseter Muscle Thickness, Chewing Efficiency and Bite Force in Edentulous Patients With Fixed and Removable Implant‐Supported Prostheses: A Cross‐Sectional Multicenter Study.” Clinical Oral Implants Research 23: 144–150. 21631592 10.1111/j.1600-0501.2011.02213.x

[cre270277-bib-0035] Najeeb, S. , M. S. Zafar , Z. Khurshid , and F. Siddiqui . 2016. “Applications of Polyetheretherketone (PEEK) in Oral Implantology and Prosthodontics.” Journal of Prosthodontic Research 60: 12–19. 26520679 10.1016/j.jpor.2015.10.001

[cre270277-bib-0036] Nazari, V. , S. Ghodsi , M. Alikhasi , M. Sahebi , and A. R. Shamshiri . 2016. “Fracture Strength of Three‐Unit Implant Supported Fixed Partial Dentures With Excessive Crown Height Fabricated From Different Materials.” Journal of Dentistry (Tehran) 13: 400–406. PMC5318496 28243301

[cre270277-bib-0037] Özdemir Doğan, D. , N. T. Polat , S. Polat , E. Şeker , and E. B. Gül . 2014. “Evaluation of “All‐on‐Four” Concept and Alternative Designs With 3D Finite Element Analysis Method.” Clinical Implant Dentistry and Related Research 16: 501–510. 23217013 10.1111/cid.12024

[cre270277-bib-0038] Pandey, A. , F. Durrani , S. K. Rai , et al. 2023. “Comparison Between All‐on‐Four and All‐on‐Six Treatment Concepts on Stress Distribution for Full‐Mouth Rehabilitation Using Three‐Dimensional Finite Element Analysis: A Biomechanical Study.” Journal of Indian Society of Periodontology 27: 180–188. 37152467 10.4103/jisp.jisp_278_22 PMC10159094

[cre270277-bib-0039] Passaretti, A. , G. Petroni , G. Miracolo , V. Savoia , A. Perpetuini , and A. Cicconetti . 2018. “Metal Free, Full Arch, Fixed Prosthesis for Edentulous Mandible Rehabilitation on Four Implants.” Journal of Prosthodontic Research 62: 264–267. 29223315 10.1016/j.jpor.2017.10.002

[cre270277-bib-0040] Pjetursson, B. E. , D. Thoma , R. Jung , M. Zwahlen , and A. Zembic . 2012. “A Systematic Review of the Survival and Complication Rates of Implant‐Supported Fixed Dental Prostheses (FDPs) After a Mean Observation Period of at Least 5 Years.” Clinical Oral Implants Research 23: 22–38. 10.1111/j.1600-0501.2012.02546.x 23062125

[cre270277-bib-0041] Ricciardi Coppedê, A. , A. C. Lapria Faria , M. G. Chiarello de Mattos , R. C. Silveira Rodrigues , J. A. Shibli , and R. Faria Ribeiro . 2013. “Mechanical Comparison of Experimental Conical‐Head Abutment Screws With Conventional Flat‐Head Abutment Screws for External‐Hex and Internal Tri‐Channel Implant Connections: An In Vitro Evaluation of Loosening Torque.” International Journal of Oral & Maxillofacial Implants 28: e321–e329. 24278937 10.11607/jomi.3029

[cre270277-bib-0042] Rosén, A. , and G. Gynther . 2007. “Implant Treatment Without Bone Grafting in Edentulous Severely Resorbed Maxillas: A Long‐Term Follow‐up Study.” Journal of Oral and Maxillofacial Surgery 65: 1010–1016. 17448855 10.1016/j.joms.2006.11.023

[cre270277-bib-0043] Sailer, I. , S. Mühlemann , M. Zwahlen , C. H. F. Hämmerle , and D. Schneider . 2012. “Cemented and Screw‐Retained Implant Reconstructions: A Systematic Review of the Survival and Complication Rates.” Clinical Oral Implants Research 23: 163–201. 23062142 10.1111/j.1600-0501.2012.02538.x

[cre270277-bib-0044] Shin, H.‐M. , J. B. Huh , M. J. Yun , Y. C. Jeon , B. M. Chang , and C. M. Jeong . 2014. “Influence of the Implant‐Abutment Connection Design and Diameter on the Screw Joint Stability.” Journal of Advanced Prosthodontics 6: 126–132. 24843398 10.4047/jap.2014.6.2.126 PMC4024557

[cre270277-bib-0045] Silva, G. C. , J. A. Mendonça , L. R. Lopes , and J. Landre, Jr . 2010. “Stress Patterns on Implants in Prostheses Supported by Four or Six Implants: A Three‐Dimensional Finite Element Analysis.” International Journal of Oral & Maxillofacial Implants 25: 239–246. 20369081

[cre270277-bib-0046] Suzaki, N. , S. Yamaguchi , N. Hirose , et al. 2020. “Evaluation of Physical Properties of Fiber‐Reinforced Composite Resin.” Dental Materials 36: 987–996. 32546399 10.1016/j.dental.2020.04.012

[cre270277-bib-0047] Taruna, M. , B. Chittaranjan , N. Sudheer , S. Tella , and M. Abusaad . 2014. “Prosthodontic Perspective to All‐on‐4® Concept for Dental Implants.” Journal of Clinical and Diagnostic Research 8: ZE16–ZE19. 25478475 10.7860/JCDR/2014/9648.5020 PMC4253293

[cre270277-bib-0048] Topcu Ersöz, M. B. , and E. Mumcu . 2022. “Biomechanical Investigation of Maxillary Implant‐Supported Full‐Arch Prostheses Produced With Different Framework Materials: A Finite Elements Study.” Journal of Advanced Prosthodontics 14: 346. 36685790 10.4047/jap.2022.14.6.346 PMC9832146

[cre270277-bib-0049] Uesugi, T. , Y. Shimoo , M. Munakata , et al. 2024. “A Study of the Associated Risk Factors for Early Failure and the Effect of Photofunctionalisation in Full‐Arch Immediate Loading Treatment Based on the All‐on‐Four Concept.” Bioengineering 11: 223. 38534497 10.3390/bioengineering11030223 PMC10968038

